# Supplementary material for: Visible Light Photoelectrochemical Sensor for Dopamine: Determination Using Iron Vanadate Modified Electrode
Source: Molecules. 2022 Sep 28;27(19):6410. doi: 10.3390/molecules27196410 (PMC9572152; doi:10.3390/molecules27196410)
Supplement: Supplementary file 1 [file molecules-27-06410-s001.zip › molecules-1920463-supplementary.pdf]

SUPPLEMENTARY MATERIAL

Visible Light Photoelectrochemical Sensor for Dopamine: Determination Using Iron Vanadate Modified Electrode

Luan Pereira Camargo[1,2,3], Marcelo Rodrigues da Silva Pelissari[4], Paulo Rogério Catarini da Silva[5], Augusto Batagin-Neto[6], Roberta Antigo Medeiros[1,2], Marcos Antônio Dias[7], and Luiz Henrique Dall'Antonia[1,2,3,*]

[1]Departamento de Química/CCE/UEL, State University of Londrina (UEL), Londrina, PR, Brazil;

[2]Graduated Program in Chemistry, State University of Londrina (UEL), Londrina, PR, Brazil;

[3]National Institute of Science & Technology in Bioanalytic (INCTBio), Campinas, SP, Brazil;

[4]Faculdade de Engenharia, CTI, São Paulo State University (UNESP), Bauru, SP, Brazil;

[5]Departamento de Física/CCE/UEL, State University of Londrina (UEL), Londrina, PR, Brazil;

[6]Instituto de Ciências e Engenharia, São Paulo State University (UNESP), Itapeva, SP, Brazil;

[7]Departmaento de Neurocirurgia, Centro de Ciências da Saúde, State University of Londrina (UEL), Londrina, PR, Brazil;

* Correspondence: luizh@uel.br (L. H. D.)

XRD analysis

XRD measurements were performed using a PANalytical diffractometer, model X'Pert PRO MPD, with CuKa radiation (1.54060 Å), coupled to a nickel filter to reduce CuKb radiation. The applied voltage was 40 kV and the current 30 mA. The scanning range was from 10° to 80°, with a regular pitch of 0.03° s^{-1}. To the characterization of the obtained pattern, the Rietveld refinement method was carried out using the HighScore Plus software. To obtain the quality level of the structure refinements, the control parameters χ^2 and R_p were obtained through Equations 1 and 2, respectively. These parameters represent a statistical test showing how much the calculated crystallographic parameters are close to the actual values:

$$\chi^2 = \frac{\sum w_i (y_{io} - y_{ic})^2}{N - P} = \left[\frac{R_{wp}}{R_{exp}}\right]^2 \quad (1)$$

$$R_p = \frac{\sum |y_{io} - y_{ic}|}{\sum y_{io}} \quad (2)$$

According to the equations above, $w_i = 1/y_i$, y_i is the intensity measured at the i-th point, and y_{ci} is the calculated (theoretical) intensity at the i-th point. The indices o and c represent the observed and calculated intensities, respectively.

Electroactive surface area studies

To study the electroactive surface area of the electrodes, cyclic voltammetry was used (from -0.2 to 0.45 V vs. Ag/AgCl). The measurements were performed in a 6.6×10^{-4} mol L^{-1} $[Fe(CN)_6]^{3-/4-}$ + 0.1 mol L^{-1} KCl solution, at the following scan rates: 5, 10, 20, 30, 40, 50, 75 and 100 mV s^{-1}. The estimated electroactive surface area was calculated according to the Randles-Sevcik equation [1], Equation 3.

$$I_p = (2.69 \times 10^5)\, n^{3/2}\, A\, D_0^{1/2}\, c\, \upsilon^{1/2}$$

Wherein n represents the number of electrons participating in the redox reaction, A is the area of the electroactive surface area (cm^2), D is the diffusion coefficient of the $[Fe(CN)_6]^{3-/4-}$ in solution (6.20×10^{-6} cm^2 s^{-1}), C corresponds to hexacyanoferrate(III) concentration in solution (mol cm^{-3}), and υ is the potential scan rate (mV s^{-1}). The $[Fe(CN)_6]^{3-/4-}$ redox system used in this study is one of the most extensively studied redox couples in electrochemistry for electroactive surface area analysis and exhibits a heterogeneous one-electron transfer (n = 1). The oxidation process's nature was controlled by diffusion in the systems studied, as evidenced by the linear plots of the anodic peak current (I_p) versus the square root of the scanning rate ($\upsilon^{1/2}$).

Artificial cerebrospinal fluid (aCSF) preparation

The artificial cerebrospinal fluid (aCSF) was prepared before each experimental session. The solution was prepared by dissolving 0.3711 g NaCl, 0.0037 g KCl, 0.0082 g KH_2PO_4, 0.1092 g $NaHCO_3$, 0.0901 g *D*-glucose, 0.0176 g $CaCl_2.2H_2O$, and 0.0132 g $MgCl_2.6H_2O$ in 50 mL of ultra-pure water (Milli-Q®). The aCSF solution was stored at 4 °C for up to 1 week [2,3].

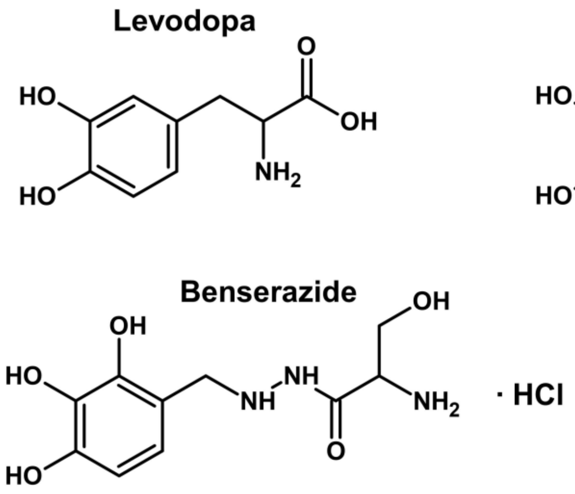

Figure S1. The structural formula of levodopa, benserazide, and dopamine molecules.

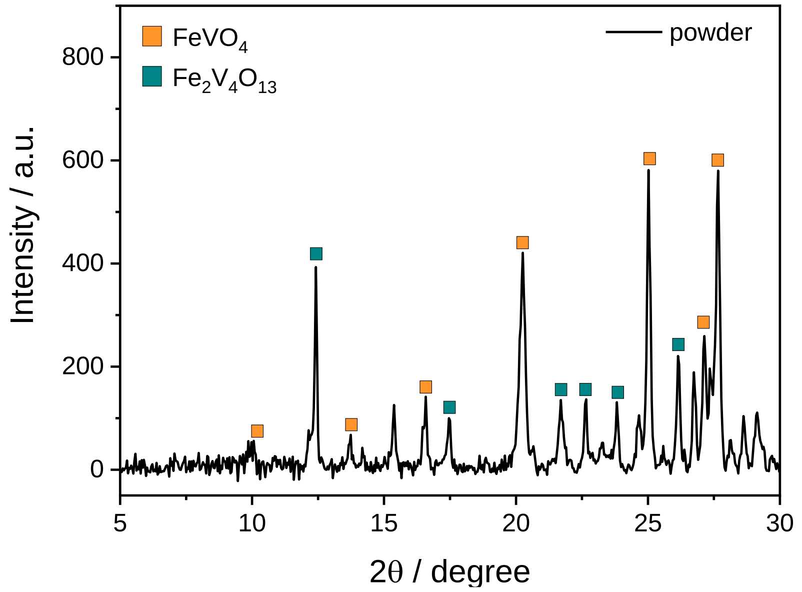

Figure S2. X-ray diffraction pattern for the powder iron vanadate sample obtained by the successive ionic adsorption and reaction (SILAR) process.

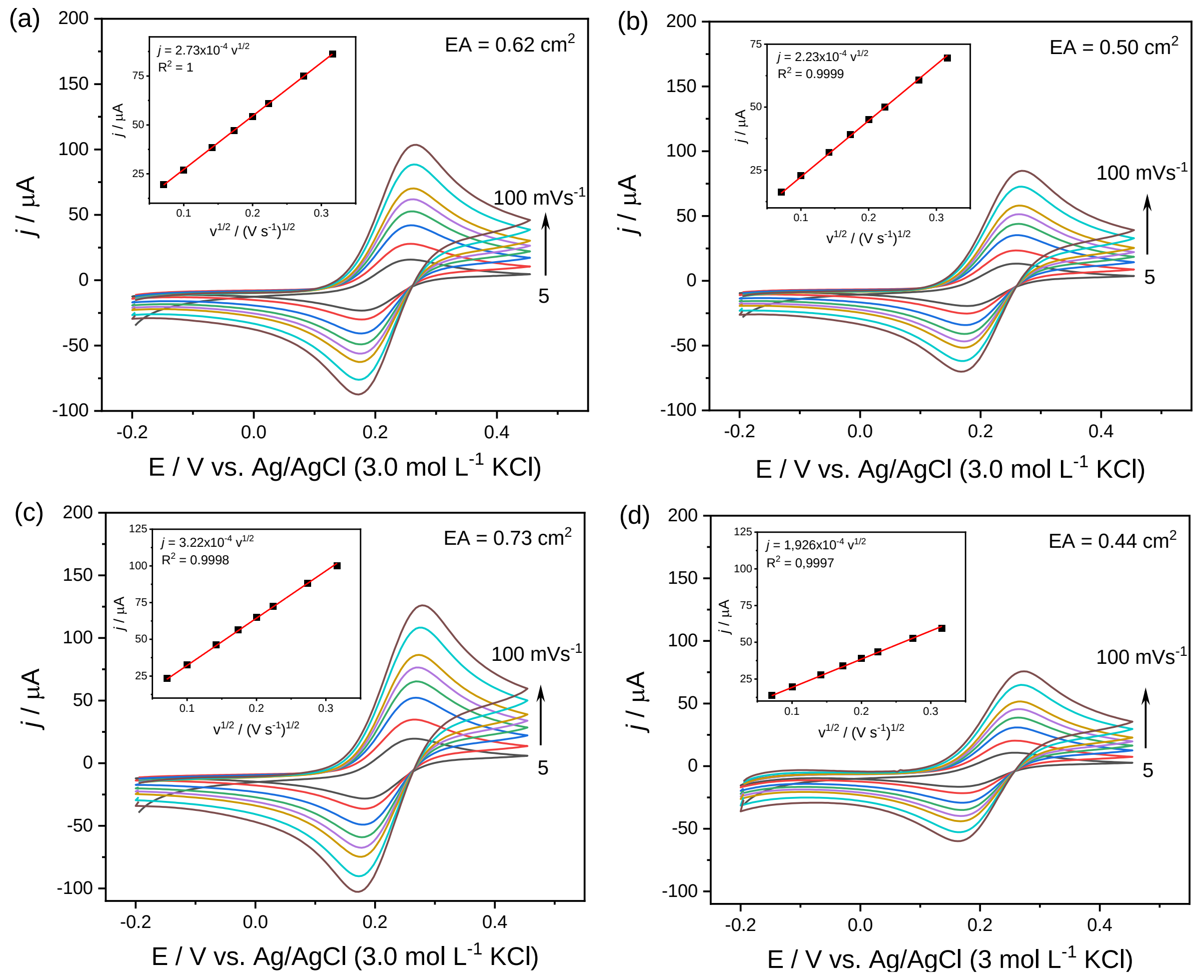

Figure S3. Cyclic voltammograms obtained in 6.6×10^{-4} mol L^{-1} $[Fe(CN)_6]^{3-/4-}$ + 0.1 mol L^{-1} KCl solution using ITO/FeVO electrodes prepared by the Successive Ionic Layer Adsorption and Reaction (SILAR) deposition process, with (**a**) 5, (**b**) 10, and (**c**) 15 layers, at the following scan rates: 5, 10, 20, 30, 40, 50, 75 and 100 mV s^{-1}. (**d**) cyclic voltammogram for the ITO conductive substrate.

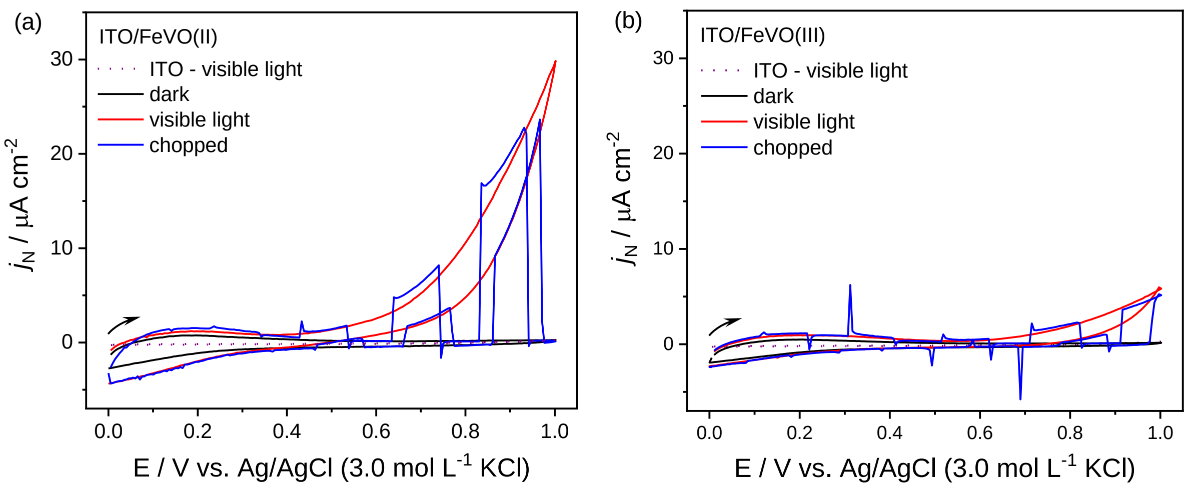

Figure S4. Photoelectrochemical measurements for the (**a**) ITO/FeVO(II), and (**b**) ITO/FeVO(III) electrodes. Cyclic voltammetry in the dark (solid black line), under continuous light illumination (solid red line), and under transient (chopped) light illumination (solid blue line) (10 s on/off) conditions. Scan rate of 10 mV s^{-1}. All measurements were performed in 0.1 mol L^{-1} Na_2SO_4 aqueous solution (pH = 5.4).

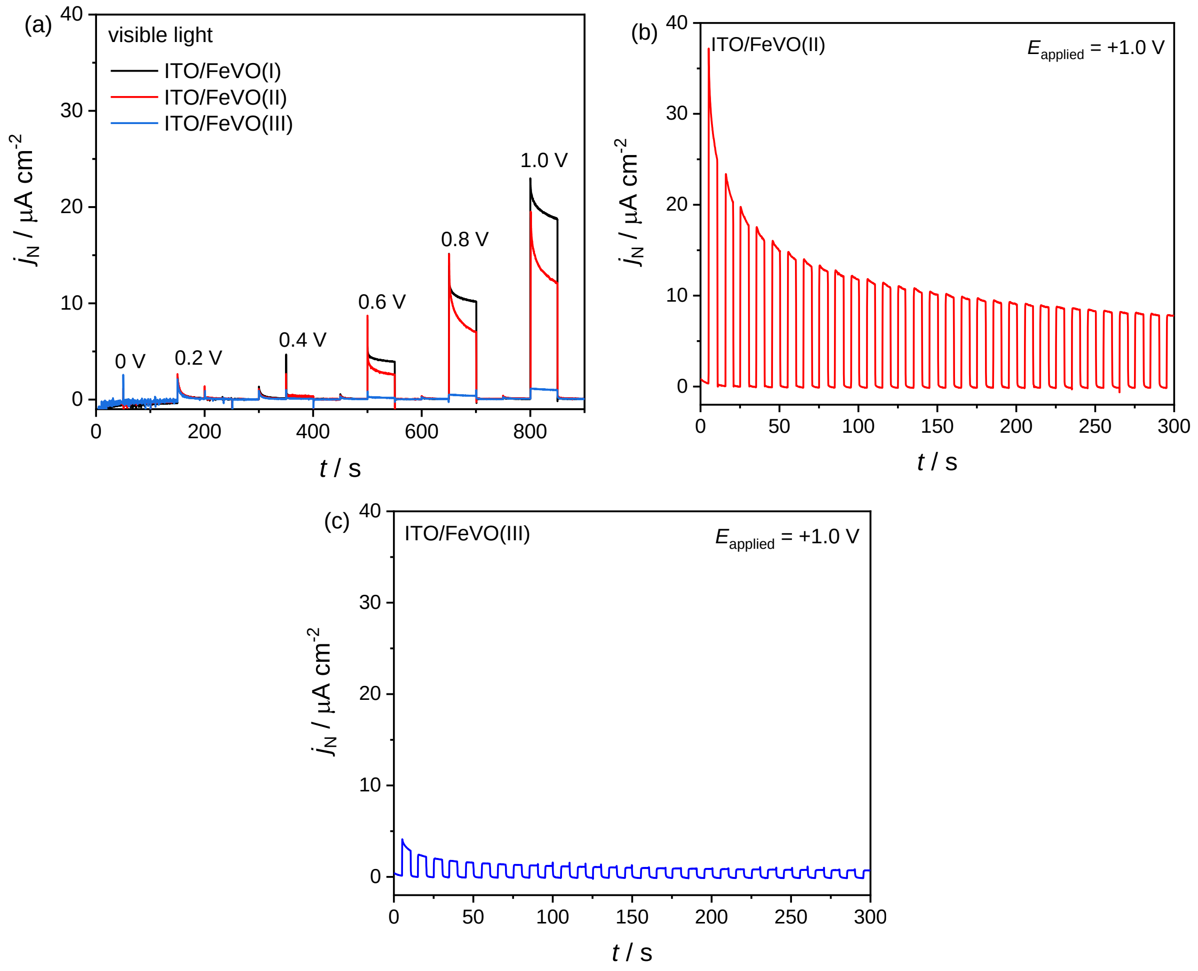

Figure S5. (**a**) Photochronoamperogram under chopped light illumination (50 s on/off), at different potentials (0, 0.2, 0.4, 0.6, 0.8, and +1.0 V vs. Ag/AgCl), for the ITO/FeVO(I), -(II), -(III) electrodes. Photochronoamperogram under chopped light illumination (5 s on/off) at +1.0 V *vs.* Ag/AgCl, for the (**b**) ITO/FeVO(II), and (**c**) ITO/FeVO(III) electrode. All measurements were performed in 0.1 mol L^{-1} Na_2SO_4 aqueous solution (pH = 5.4), and the visible light (Xe lamp) was used.

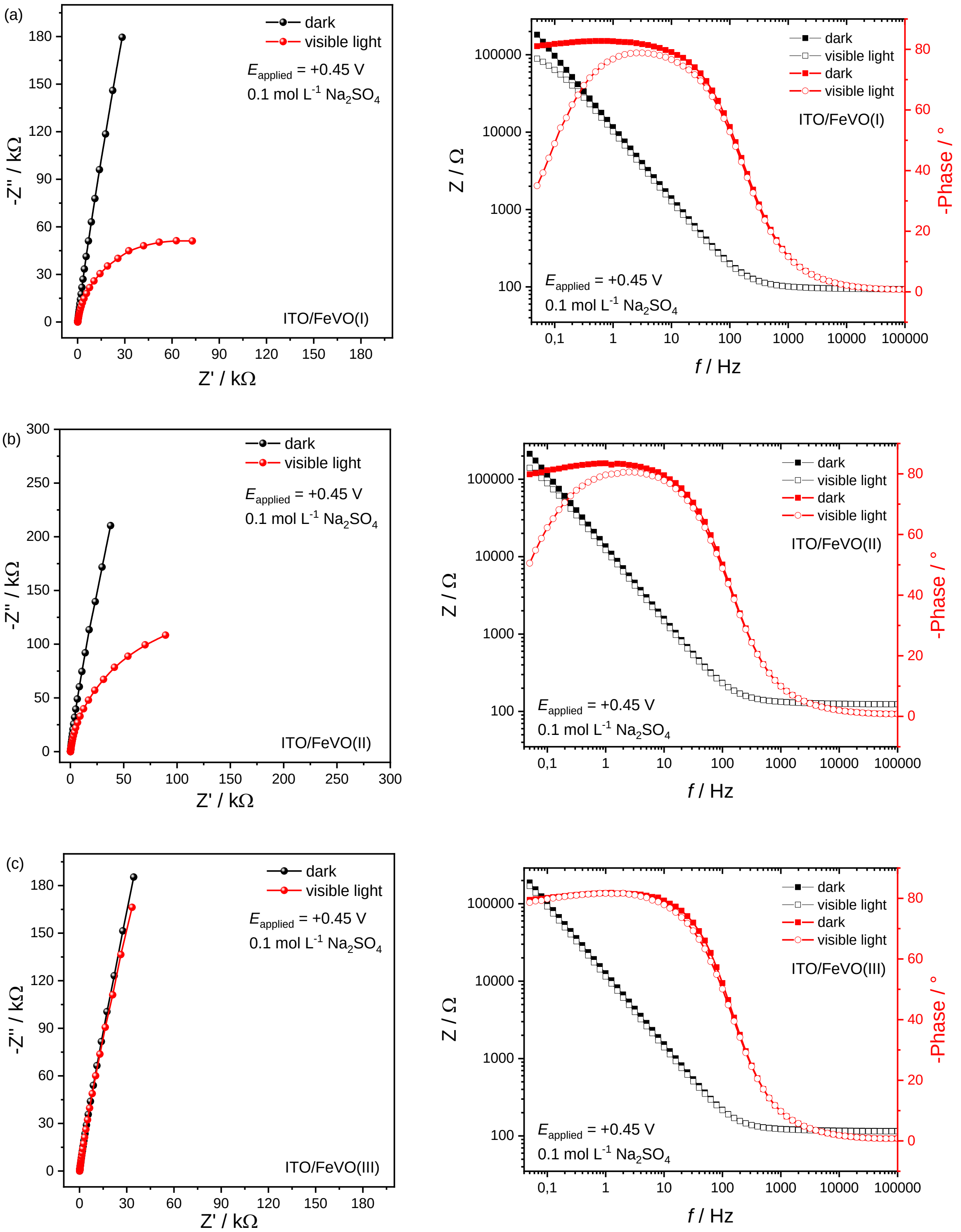

Figure S6. Nyquist and Bode plots obtained in 0.1 mol L^{-1} Na_2SO_4 for the electrochemical impedance measurements of (**a**) ITO/FeVO(I), (**b**) ITO/FeVO(II), and (**c**) ITO/FeVO(III) electrode, at +0.45 V (*vs.* Ag/AgCl).

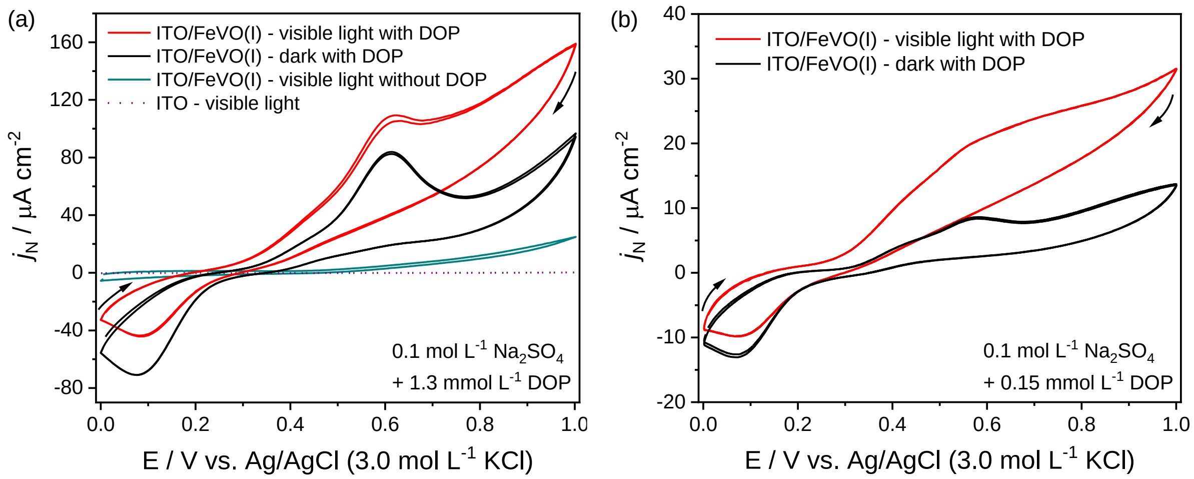

Figure S7. Cyclic voltammograms obtained in (**a**) 1.3 mmol L^{-1}, and (**b**) 0.15 mmol L^{-1} dopamine solution using the ITO/FeVO(I) electrode prepared by the Successive Ionic Layer Adsorption and Reaction (SILAR) deposition process. Supporting electrolyte: 0.1 mol L^{-1} Na_2SO_4 solution. Scan rate 20 mV s^{-1}.

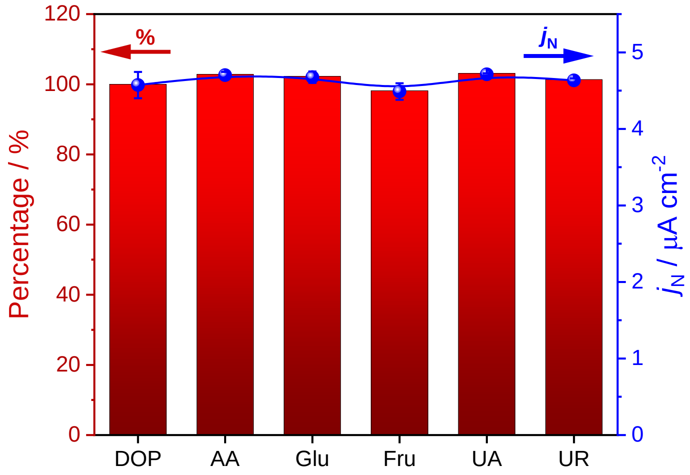

Figure S8. Photocurrent response obtained with ITO/FeVO(I) electrode during successive addition of dopamine (DOP), ascorbic acid (AA), glucose (Glu), fructose (Fru), uric acid (UA), and urea (UR). Species concentrations: 10 µmol L^{-1}; supporting electrolyte: 0.1 mol L^{-1} Na_2SO_4 solution.

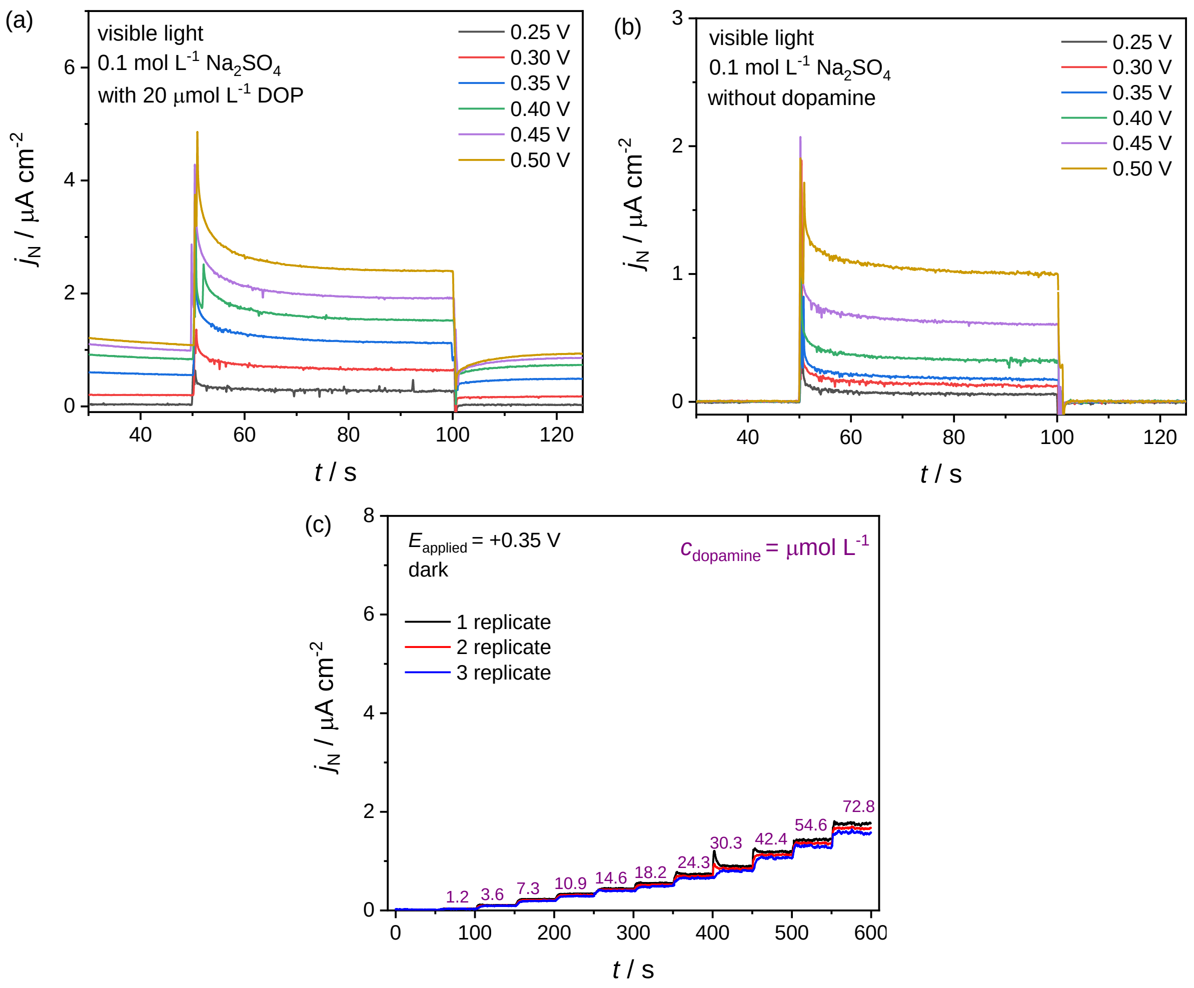

Figure S9. Photochronoamperogram of ITO/FeVO(I) electrode (**a**) with 20 µmol L^{-1} and (**b**), without dopamine, under different applied potentials (0.25, 0.30, 0.35, 0.40, 0.45, 0.50 V (*vs.* Ag/AgCl)). (**c**) The current density response was obtained for different dopamine concentrations (from 1.21 to 72.77 µmol L^{-1}). Measurement was performed in triplicate and in the absence of visible light.

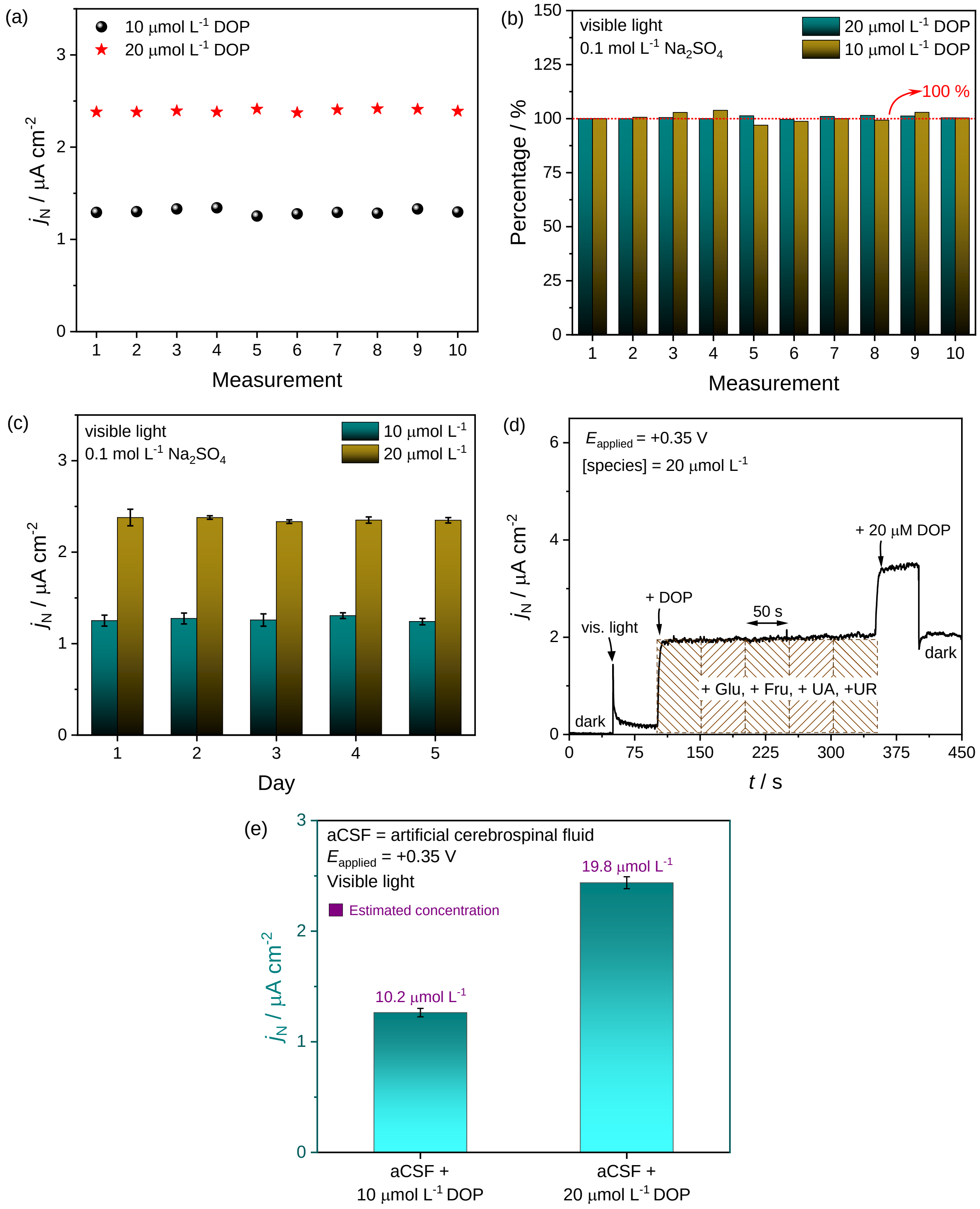

Figure S10. Photocurrent response of ITO/FeVO(I) electrode during (**a-b**) intra- and (**c**) inter-day repeatability studies. Measurements performed with 10 and 20 µmol L^{-1} dopamine solutions. (**d**) Chronoamperogram for the ITO/FeVO(I) electrode during successive addition of dopamine (DOP), glucose (Glu), fructose (Fru), uric acid (UA), and urea (UR). Interferent measurement performed with the potential application of +0.35 V (*vs.* Ag/AgCl) and either presence or absence of visible

light. Supporting electrolyte: 0.1 mol L^{-1} Na_2SO_4. (**e**) Photocurrent response and estimated concentration for the 10 and 20 µmol L^{-1} dopamine solution in artificial cerebrospinal fluid (aCSF).

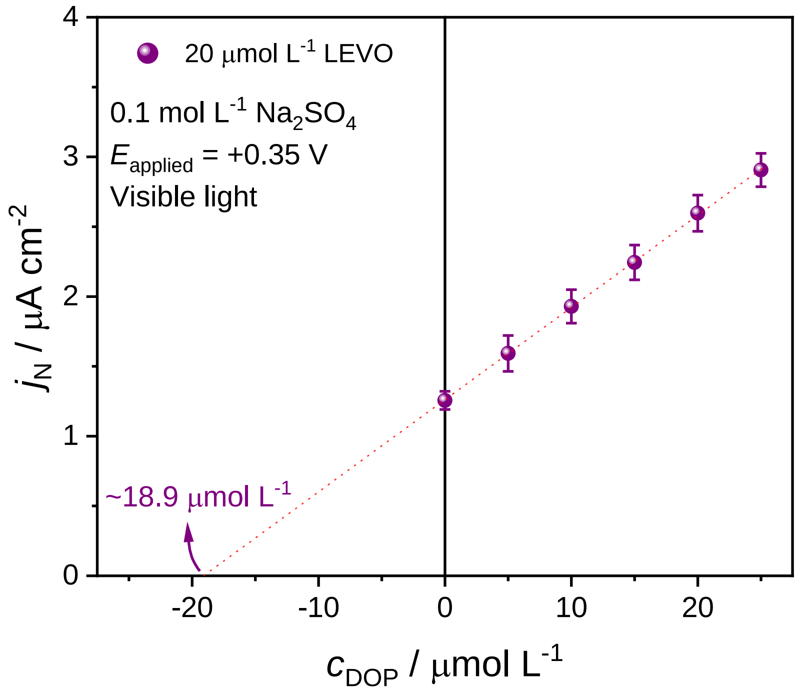

Figure S11. Photocurrent response for commercial drug sample, contained levodopa, using the standard addition method. Measurement was performed in triplicate with the addition of 150 µL of the diluted sample, followed by successive addition of dopamine solution (5-25 µmol L^{-1}). 0.1 mol L^{-1} Na_2SO_4 solution was used as the supporting electrolyte.

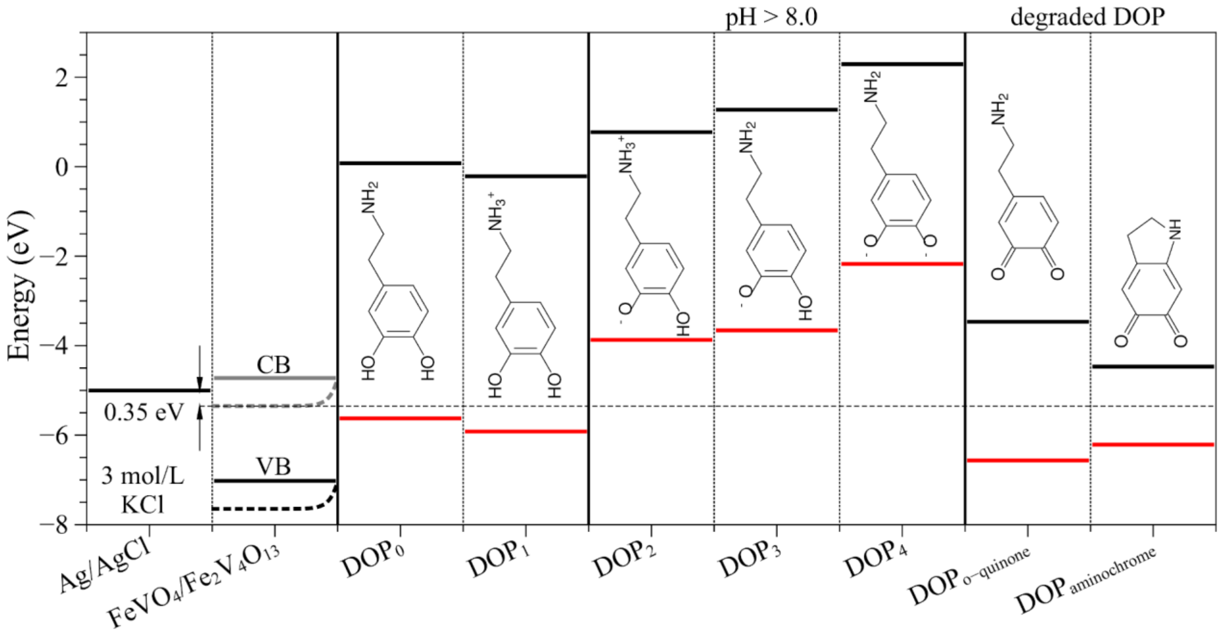

Figure S12. Relative alignment between the frontier electronic levels of electrodes and dopamine species.

Figure S12 presents the relative alignment between frontier electronic levels of reference/working electrodes and dopamine species. DOP_0 and DOP_1 are the dominant species at the experimental conditions (pH ~ 5.4), while DOP_2-DOP_4 are expected only in alkaline solutions. $DOP_{o\text{-quinone}}$ and $DOP_{\text{aminochrome}}$ represent byproducts expected from DOP oxidation. Given the levels position, the oxidation process noticed at +0.35 V (*vs*. Ag/AgCl) can be linked to DOP_0. The oxidation peak at ~0.6 V (*vs*. Ag/AgCl) (Figure S7a,b) can be ascribed to DOP_1 or degraded $DOP_{\text{aminochrome}}$.

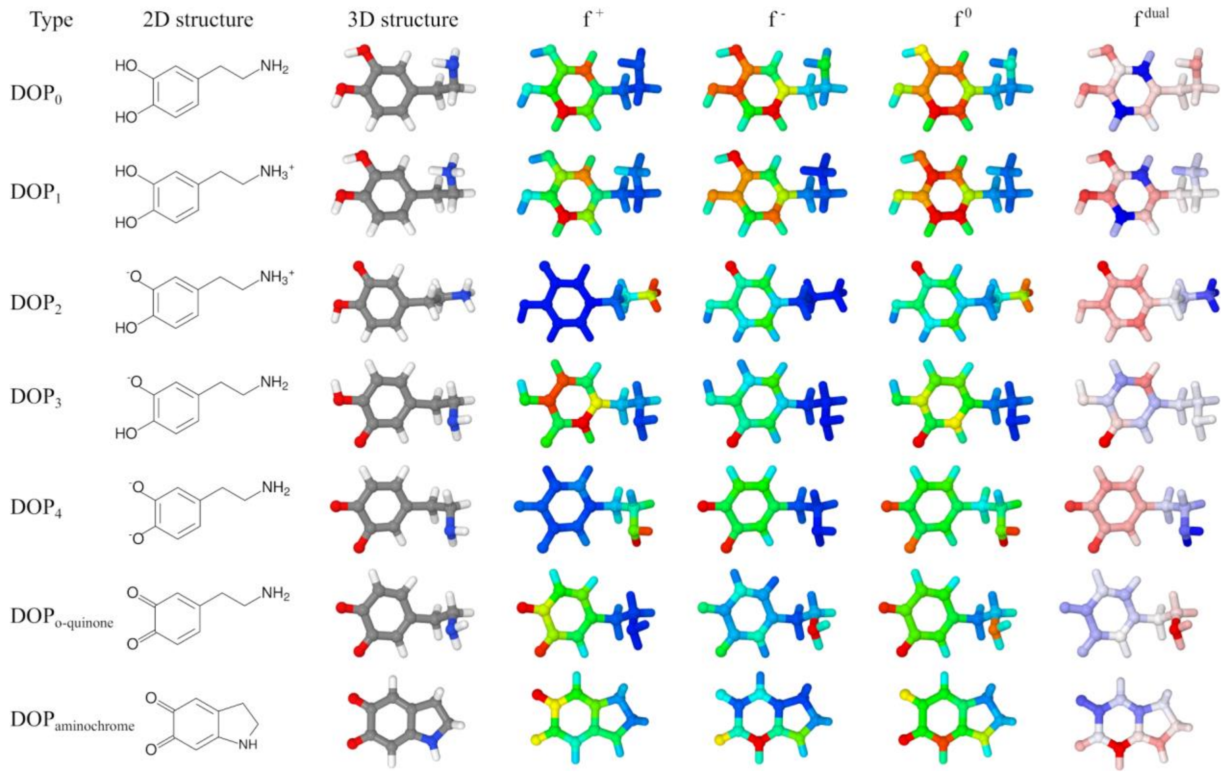

Figure S13. CAFI colored maps of DOP species. Red and blue regions indicate reactive and inert regions for reactions towards nucleophiles (f^{+}), electrophiles (f^{-}) and free radicals (f^{0}) or the position of electron acceptor and electron donor sites (for f^{dual}).

Figure S13 illustrates the local reactivity of the compounds via colored condensed-to-atoms Fukui indexes (CAFI: f^{+}, f^{-} and f^{0}) and dual descriptor $f^{dual} = f^{+} - f^{-}$. Red and blue regions define reactive and inert molecular sites. Given their definition, f^{+}, f^{-} and f^{0} define the tendency of the compounds to interact with nucleophiles, electrophiles, and free radicals. In other words, high values of f^{+} and f^{-} (red regions) indicate which sites are prone to receive or donate electrons, while f^{0} indicate the mean values ($½(f^{+}+f^{-})$). f^{dual}, in turn, assumes positive values (red regions) on molecular sites that are better electron acceptors than donors and are negative (blue) on sites with dominant donor features. The results indicate that DOP_1 and DOP_2 tend to oxidize (high values of f^{-} and negative f^{dual}) via ring-electrode interactions.

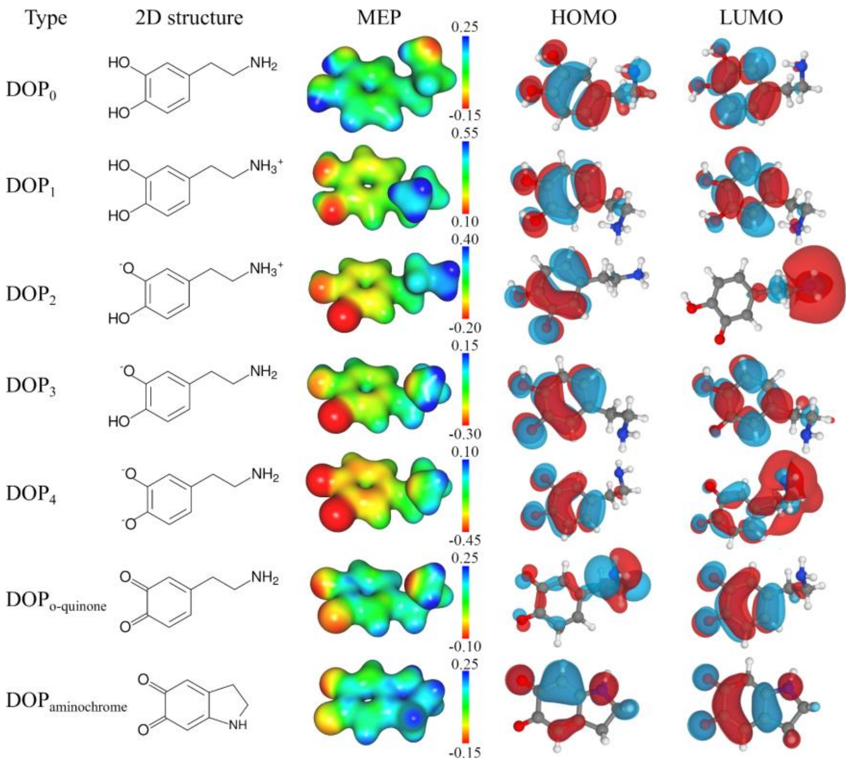

Figure S14. MEP colored maps and FMOs of DOP species.

Figure S14 presents the colored molecular electrostatic potential (MEP) and spatial distribution of the frontier Kohn-Sham molecular orbitals (FMO – HOMO and LUMO) of DOP species. Red and blue regions define negative (or less positive) and (more) positive electrostatic potential.

Similar MEPs/FMO distribution have already been reported in the literature, in particular for DOP_0 [4,5], and are centered in the ring (as already suggested by CAFI). In fact, the relevance of stacking process (i.e., interaction of DOP with the active surface of the electrode via its ring) for DOP oxidation have already been reported in the literature with other electrodes [6] and are compatible with our results (from CAFI and FMO analyses), reinforcing the relevance of DOP-to-electrode charge transfer via the resonant ring.

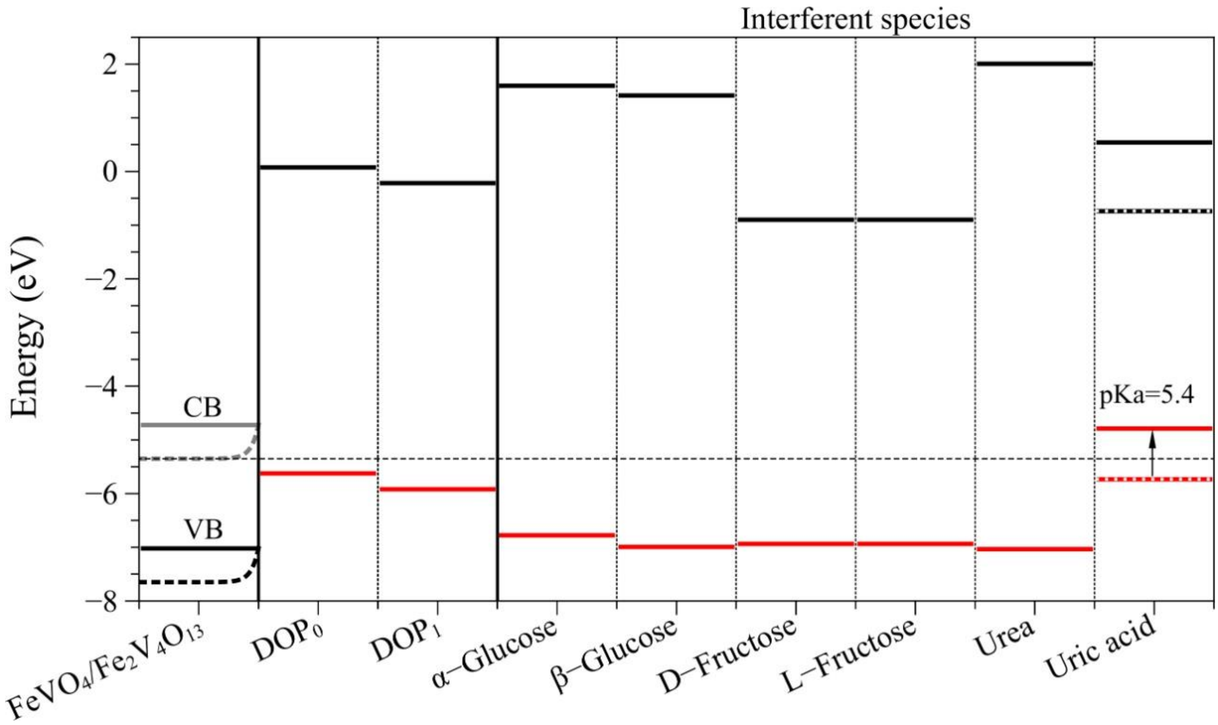

Figure S15. Relative alignment between the frontier electronic levels of electrodes and organic species.

Figure S15 presents the relative alignment between frontier electronic levels of reference/working electrodes, DOP_0, DOP_1 and distinct interfering species. Note that, at the experimental conditions, there is no favorable alignments effective charge transfer processes between the organic molecules and the ITO/FeVO(I) electrode, confirming the interference study.

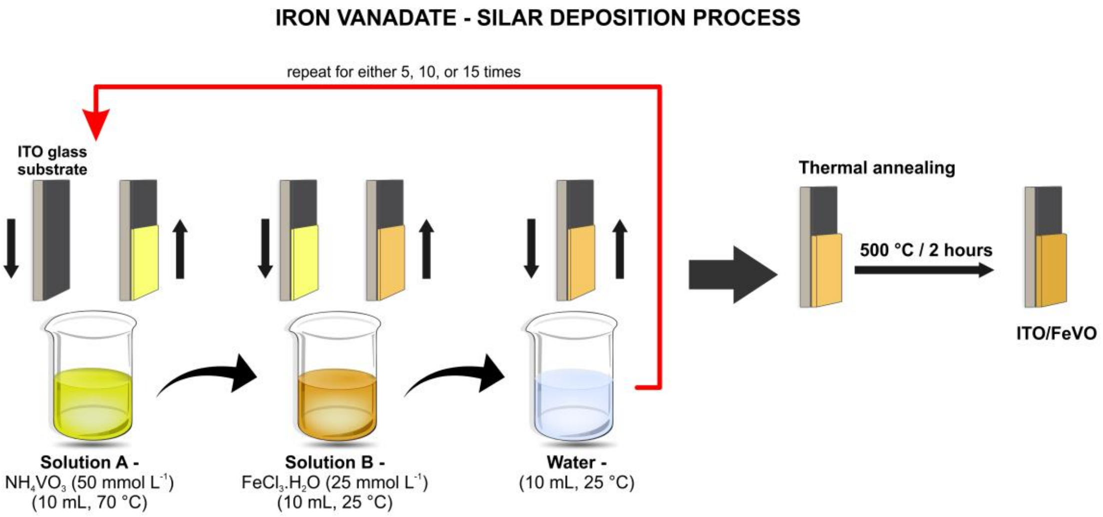

Figure S16. Schematic diagram showing details of the Successive Ionic Layer Adsorption and Reaction (SILAR) deposition process. ITO surface area was limited to 1 cm^2, and for each solution (*A*, *B*, and water solutions), the immersion was maintained for 5 minutes.

Table S1. Summary of the electrochemical impedance spectroscopy results, for the ITO/FeVO electrodes obtained by the SILAR process. Measurements obtained under light (visible light) and dark condition. Applied potential: +0.45 V (*vs.* Ag/AgCl). Nova 2.1.5 software report.

**Electrode – condition**	**Parameter**	**Value**	**Estimated error / %**
ITO/FeVO(I) – dark	R_s / Ω	95.5	0.2
	R_{ct} / kΩ	5525.4	13.9
	CPE / μF	16	03
ITO/FeVO(I) – visible light	R_s / Ω	94.7	0.3
	R_{ct} / kΩ	119.0	0.9
	CPE / μF	18	0.4
ITO/FeVO(II) – dark	R_s / Ω	127.1	0.4
	R_{ct} / kΩ	2561.2	13.5
	CPE / μF	13	0.5
ITO/FeVO(II) – visible light	R_s / Ω	126.1	0.3
	R_{ct} / kΩ	257.0	1.7
	CPE / μF	15	0.5
ITO/FeVO(III) – dark	R_s / Ω	116.2	0.4
	R_{ct} / kΩ	2224.7	13.8
	CPE / μF	16	0.5
ITO/FeVO(III) – visible light	R_s / Ω	116.2	0.2
	R_{ct} / kΩ	2160.0	9.3
	CPE / μF	16	0.3

R_s = solution resistance. R_{ct} = resistance of charge transference process. CPE = capacitance of the double electrical layer.

Table S2. Results for dopamine standard recovery from artificial cerebrospinal fluid samples.

	Sample			
	Added / µmol L^{-1}	j_N / µA cm^{-2} [1]	Found / µmol L^{-1}	Recovery / %
artificial	10.0	1.26 (±0.04)	10.24 (±0.15)	102.4
cerebrospinal fluid	20.0	2.44 (±0.05)	19.75 (±0.11)	98.7

[1] Photocurrent density (j_N) responses for chronoamperometry measurements at +0.35 V (*vs.* Ag/AgCl).

References

1. Allen, J.B.; Larry, R.F.; Bard, A.J.; Faulkner, L.R. *Electrochemical Methods: Fundamentals and Applications*; 2nd ed.; Wiley: New York, 2001; ISBN 978-0-471-04372-0.
2. Fishman, R.A. Physiology of the Cerebrospinal Fluid. *Arch. Neurol.* **1968**, *18*, 459–460, doi:10.1001/archneur.1968.00470340145021.
3. Davson, H. *Physiology of the Cerebrospinal Fluid*; Harcourt Brace/Churchill Livingstone: London, 1967; ISBN 978-0700012909.
4. Soltani; Baei A DFT Study on Structure and Electronic Properties of BN Nanostructures Adsorbed with Dopamine. *Computation* **2019**, *7*, 61, doi:10.3390/computation7040061.
5. Rajendrachari, S.; Kudur Jayaprakash, G.; Pandith, A.; Karaoglanli, A.C.; Uzun, O. Electrocatalytic Investigation by Improving the Charge Kinetics between Carbon Electrodes and Dopamine Using Bio-Synthesized CuO Nanoparticles. *Catalysts* **2022**, *12*, 994, doi:10.3390/catal12090994.
6. Rossi-Fernández, A.C.; Meier, L.A.; Castellani, N.J. Neutral and Zwitterionic Dopamine Species Adsorbed on Silver Surfaces: A DFT Investigation of Interaction Mechanism. *Int. J. Quantum Chem.* **2019**, *119*, e25817, doi:10.1002/qua.25817.
